# Gamified E-learning in medical terminology: the TERMInator tool

**DOI:** 10.1186/s12909-020-02204-3

**Published:** 2020-08-28

**Authors:** Anna-Henrikje Seidlein, Hartmut Bettin, Philipp Franikowski, Sabine Salloch

**Affiliations:** 1 Institute of Ethics and History of Medicine, University Medicine Greifswald, Ellernholzstr. 1‐2, 17487 Greifswald, Germany; 2 Department of General Psychology II, Institute for Psychology, University of Greifswald, Franz-Mehring-Str. 47, 17489 Greifswald, Germany; 3 Institute for History, Ethics and Philosophy of Medicine, Hannover Medical School, Carl-Neuberg-Str. 1, 30625 Hannover, Germany

**Keywords:** Medical terminology, Gamification, E-learning, Higher education, Game elements

## Abstract

****Background**:**

Proficiency in medical terminology is an essential competence of physicians which ensures reliable and unambiguous communication in everyday clinical practice. The attendance of a course on medical terminology is mandatory for human and dental medicine students in Germany. Students’ prerequisites when entering the course are diverse and the key learning objectives are achieved to a varying degree.

****Methods**:**

A new learning space, the “TERMInator”, was developed at the University Medicine Greifswald to meet the medical students’ individual learning needs better. The interactive e-learning course serves as a supplement to the seminars, lectures and tutorials to rehearse and practically apply the course contents at an individual pace. It uses gamification elements and is supplied via the learning platform Moodle. The TERMInator was pilot implemented in two consecutive winter terms (2018/19, 2019/20) and comprehensively evaluated based on the general course evaluations and an anonymous questionnaire covering aspects of content, layout and user friendliness of the TERMInator and questions concerning the students’ learning preferences.

****Results**:**

The TERMInator was rated very positively overall, which was also fed back to the lecturers during the classes. Students appreciate the new e-learning tool greatly and stress that the TERMInator should be further expanded. The handling of the TERMInator was considered to be very easy and, therefore, almost no training time was needed. The tasks were easy to understand and considered a good supplement to the seminar contents. The extent and quality of the images were seen rather critically. The students’ learning strategies differ. Although e-learning options were generally rated as very important, student tutorials were considered by far the most important.

****Conclusions**:**

Medical terminology classes are characterised by heterogeneous learning groups and a high workload within a short time, which can lead to major challenges for the teaching staff. Complementary gamified e-learning tools are promising in view of the students’ different knowledge levels and changing learning behaviour.

**Trial registration:**

Not applicable.

## Background

Proficiency in medical terminology is an essential competence of physicians which ensures reliable and unambiguous communication in everyday clinical practice. The adequate use of medical terminology, for example, in medical records and shift handovers, is, therefore, essential for patient safety and effective workflows [1]. On the other hand, medical terminology plays a role in discussions with patients concerning their diagnosis, therapy and prognosis. Patients’ health literacy has increased and so has their knowledge of medical terminology and its usage [2]. However, key communicative competences of physicians still include the explanation of medical terms and their translation into a comprehensible language.

A mandatory course on medical terminology is included in the German Medical Licensure Act for Physicians (“*Ärztliche Approbationsordnung*”) in the preclinical part of the curriculum. The course bears specific challenges for university lecturers in educational practice due to the often heterogeneous groups of students in their initial study phase: Firstly, while some medical students benefit from their proficiency in Latin (and, to a lesser extent, Greek) gained in high school, others cannot draw on previously gained knowledge as Latin is an elective subject at German high schools. Secondly, while some medical students are German native speakers, for others, German is a foreign language, which makes it particularly challenging for them to deal with the extensive Greco-Latin technical vocabulary in medicine and its corresponding German terms. Thirdly, there are students who have already completed a vocational training before starting their medical studies (e.g. nurses, paramedics), whereas other students start their medical studies immediately after high school. Consequently, students vary in age and have a wide range of language competencies and practical experience which influences their learning behaviour and success. Individual transitions and biographies are of particular importance for the subject of medical terminology and can facilitate or complicate learning.

Medical terminology is taught heterogeneously at German universities as there is no nationwide curriculum or framework: There are purely self-study or e-learning courses combined with tutorials and/or consultation hours with lecturers and seminars with tutorials or lectures. Textbooks or lecture notes are currently mainly used as teaching aids. However, students’ preferences for learning materials have changed and alternatives to textbooks are becoming increasingly important. Online tools and materials are such alternatives, which are attractive as they cost little [3], are up-to-date and can be easily maintained by their creators. Instructors need to consider such changes in students’ learning preferences and adapt their teaching style and materials accordingly. Learning management systems in higher education facilitate the creation of learning tools and materials by the instructors; they might help to meet the students’ needs for up-to-date online material which is easy to access and accommodates their learning habits. Innovative learning aids for students regarding medical terminology have so far used, for example, mnemonics [4] and word matching games [5]. Such exercise materials with a playful incentive offer the opportunity of actively engaging with the course content differently and, thus, deepen students’ knowledge and understanding in a sustainable way [6, 7]. The use of typical game design elements outside of an actual gaming context is referred to as “gamification” [8]. Gamified learning has the potential to improve the students’ attitude towards the learning content, their engagement during the course and overall achievements [6, 9].

Each year about 220 first term students in human and dental medicine at the University Medicine Greifswald (Greifswald, Germany) pass a seven-week course in medical terminology consisting of lectures and face-to-face seminars given by university lecturers. The classes are accompanied by optional tutorials taught by medical students from higher terms. Key learning objectives refer to a sound knowledge of the basics of the Latin language as far as relevant for medical terminology (e.g. declensions of nominative and genitive). Furthermore, the course enables the students to analyse, understand and explain medical compounds by anatomising them into their components (prefix, root word, suffix). The course generally aims at the students becoming more confident in anatomical nomenclature and basic clinical vocabulary, being able to pronounce medical terms correctly and use them in a grammatically correct way. Moreover, the students learn about the historical contexts (etymological roots) that have influenced the development of the terms and their present use and meaning.

As a consequence of the students’ highly individual prerequisites when entering the course, the key learning objectives are achieved to a varying extent. In order to overcome major differences in learning outcomes, potential improvements were identified satisfying the individual needs of the students more efficiently with the resources currently available at the University Medicine Greifswald. In the light of the specific challenges described above, an overview of publicly available e-learning offers and tools on medical terminology in the German language was developed. A small number of apps were found, but these were partly of questionable quality and caused additional costs for the students. Due to the deficiencies and weaknesses of existing tools, the teaching staff decided to create a separate, suitable learning tool that is in accordance with the students’ learning preferences and facilitates achieving the key objectives of the medical terminology course. These aims are addressed by improving the motivation and individual learning outcomes with the assistance of a learning environment created by an interactive and playful tool, supplied via the learning platform Moodle (Modular Object-Oriented Dynamic Learning Environment). Moodle is an open-source learning management system that provides instructors with ready-to-use tools to develop and implement online courses and learning aids. Moodle follows a constructivist approach towards learning and teaching [10]. Learning within the constructivist paradigm is based on four main characteristics: the importance of previous knowledge for learning, the construction of meaning by the learners, the key role of social interaction and the need for authentic learning tasks [11].

As a result of this analysis, this article presents a new and additional learning space that has been created and describes its pilot implementation and evaluation of the students’ acceptance at the University Medicine Greifswald in two consecutive winter terms.

## Methods

**Fig. 1:**
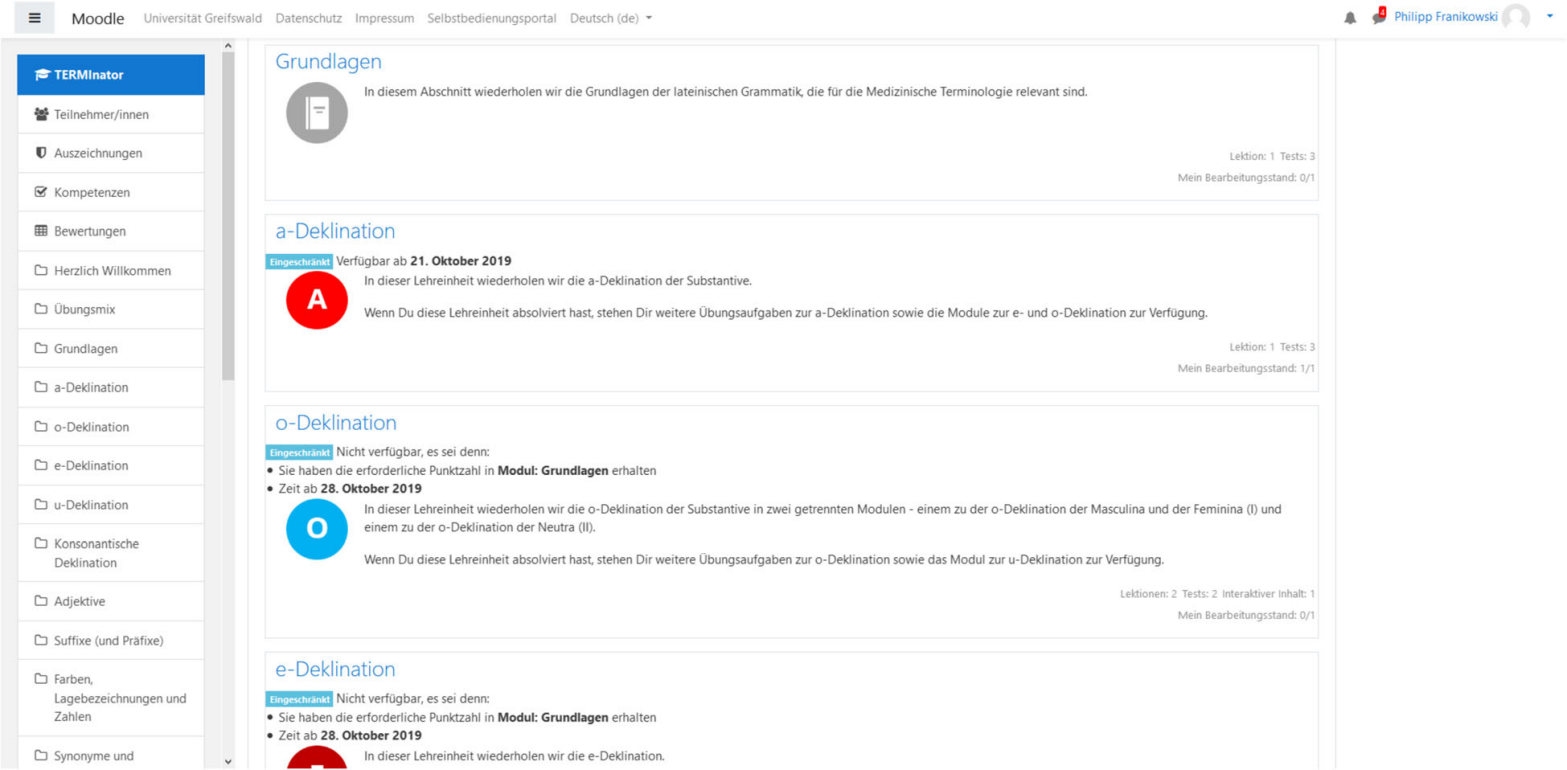
Course overview. Only unlocked courses are visible and accessible

An e-learning tool, called “TERMInator” (“TERMI” refers to medical terminology and, in addition, the complete term derives from the Latin word *“terminare”* with the meaning “to finish”), was developed for the introductory level medical students as a supplement to the seminars, lectures and tutorials to rehearse and apply the course contents practically at an individual pace. The “TERMInator” currently consists of seven modules (Fig. 1), each reflecting the contents of the respective face-to-face classes:

- “Basics” (e.g. pronunciation, emphasis)
- “Latin nouns and their declension”
- “Latin adjectives and their declension”
- “Colours, numerals, body structure”
- “Word formation” (e.g. understanding and creating complex compounds with prefixes, suffixes and roots words)
- “Synonyms and antonyms”
- “History of medical terminology”

**Fig. 2:**
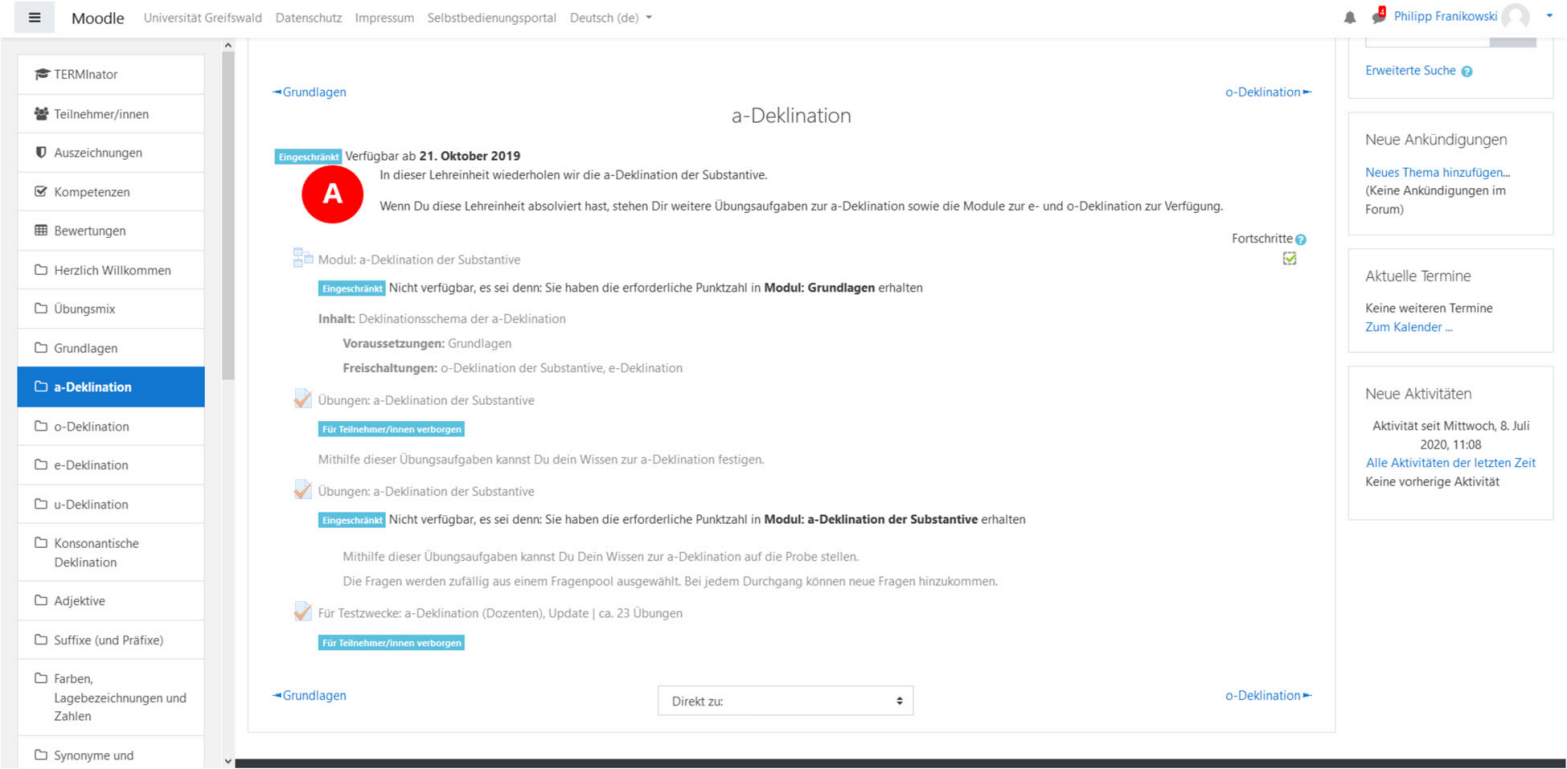
Section content overview. Only unlocked modules and exercises are visible and accessible

**Fig. 3:**
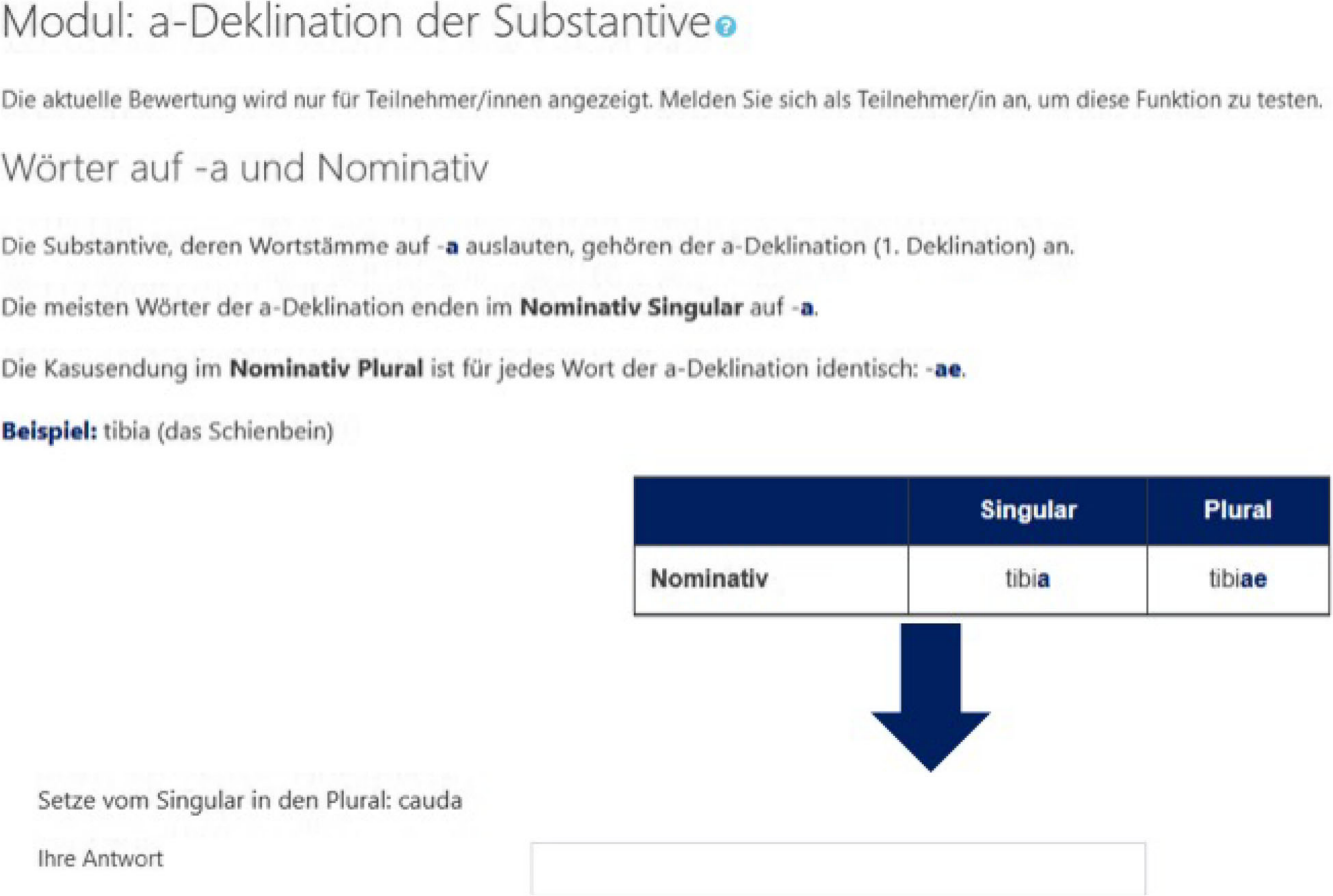
Example of a rehearsal block. Top: rule display; bottom: short exercise

The largest share of the modules consists of two consecutively available (“unlocked”) blocks, namely, a rehearsal block and an exercise block (Figs. 2 and 3).

**Fig. 4:**
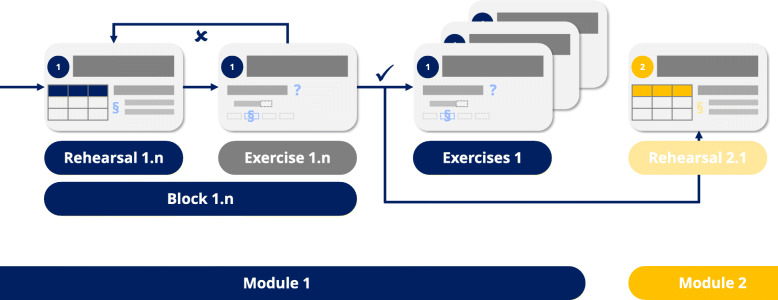
Modular structure of the TERMInator. As the final block is completed, an exercise pool rehearsing the whole module content and the next module is unlocked

In the rehearsal block, the relevant rules introduced in the seminars are rehearsed step-by-step. Each step of that recapitulation is followed by a short exercise requiring the students to apply the respective rules. If the students apply the rules correctly, the next rehearsal step is unlocked. Otherwise, students return to the previous rule display. A broader variety of exercises for recapitulation is offered in the exercise block, tailored to prepare the students for the pending exams. Those exercise blocks are only unlocked to the students after they have completed the respective rehearsal block. The module flow is outlined in Fig. 4. While most of the modules follow that structure, some of the latter topics are not covered with a rehearsal block as they depend mostly on vocabulary or are based on contents of the previous modules.

**Fig. 5:**
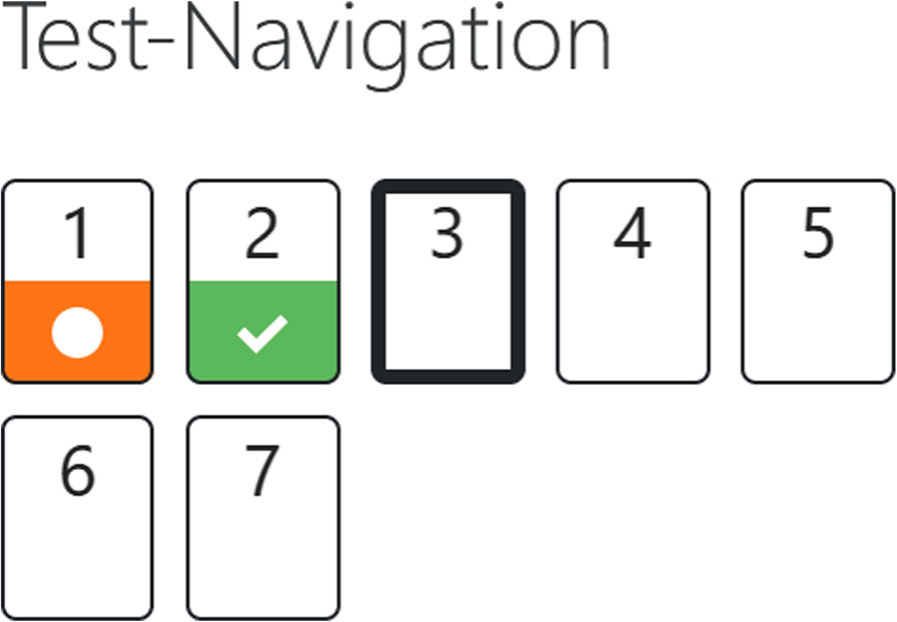
Progress bars. Progression with evaluation presented alongside the exercises

**Fig. 6:**
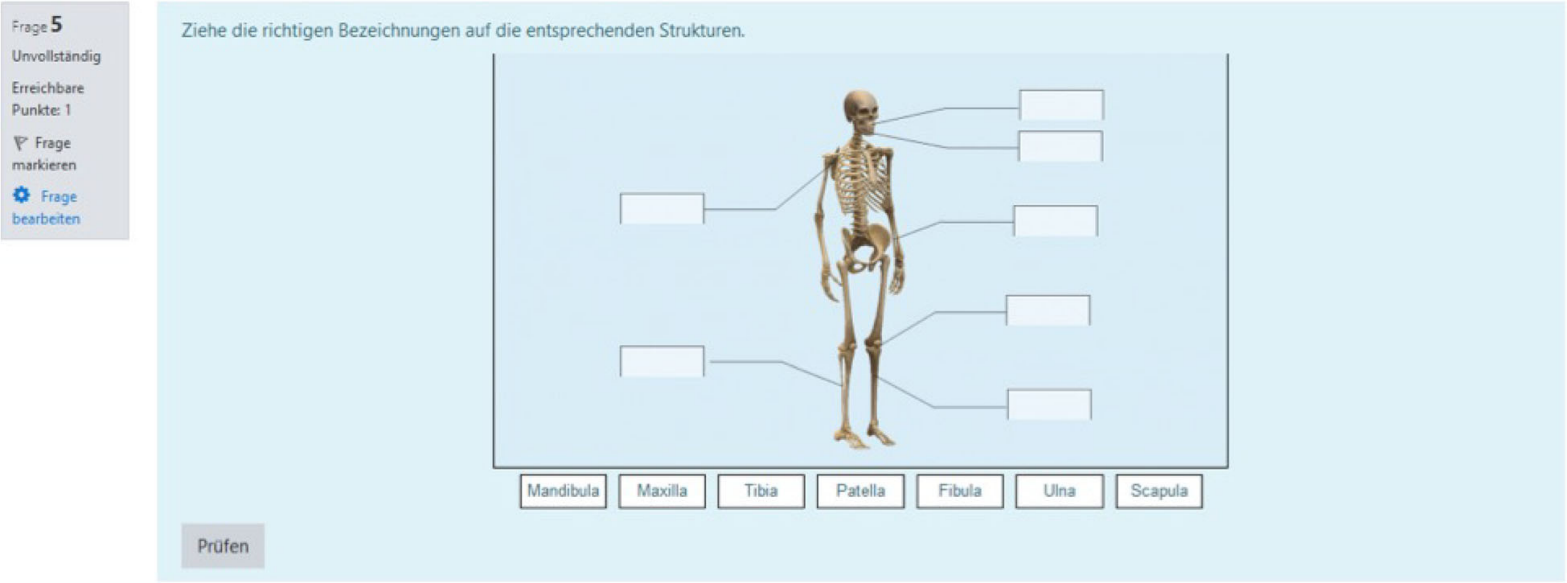
Drag & drop. Drag & drop pictorial task example

**Fig. 7:**
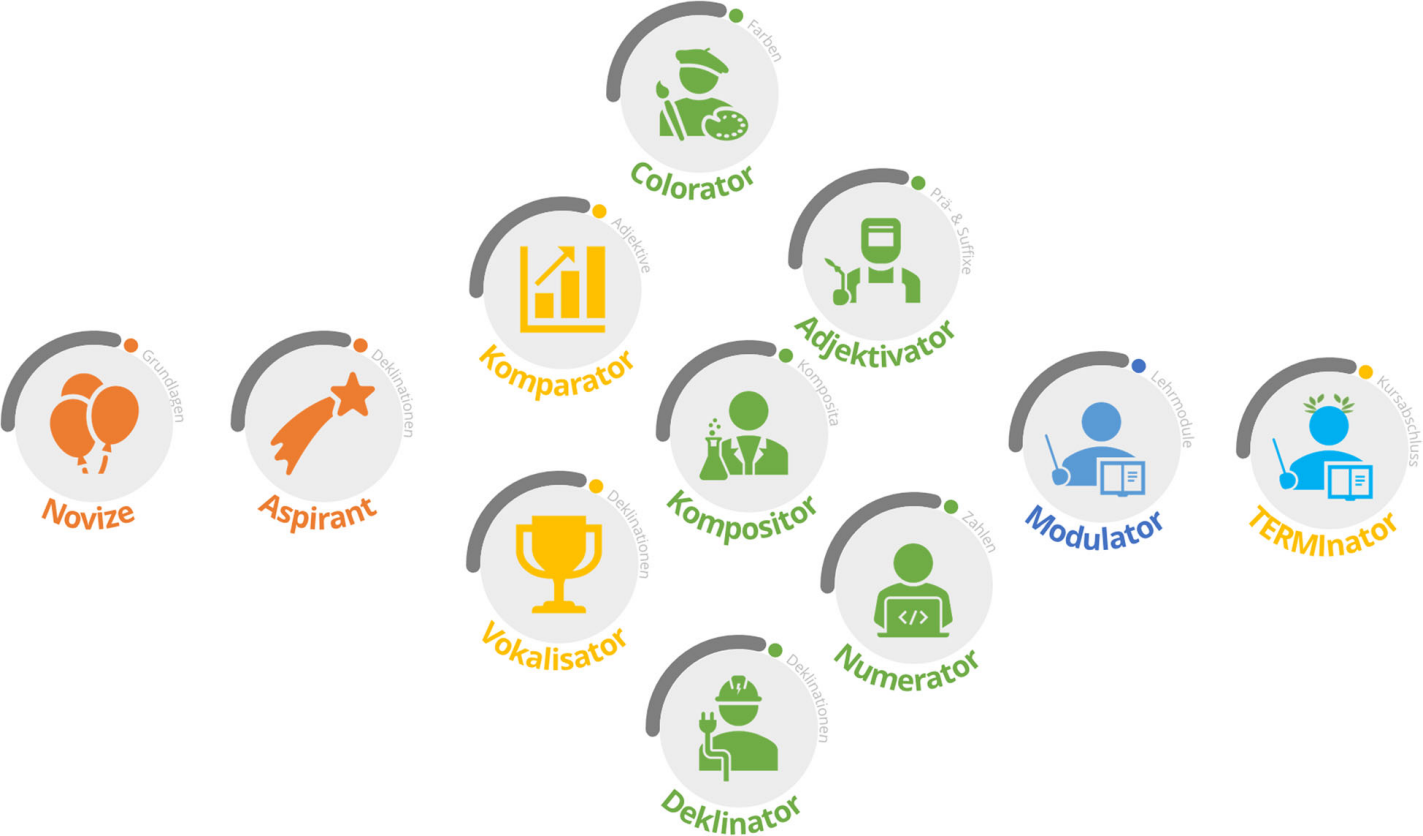
Possible certificates for the users. Awards unlocked after meeting certain criteria (e.g. completing all declination modules)

**Fig. 8:**
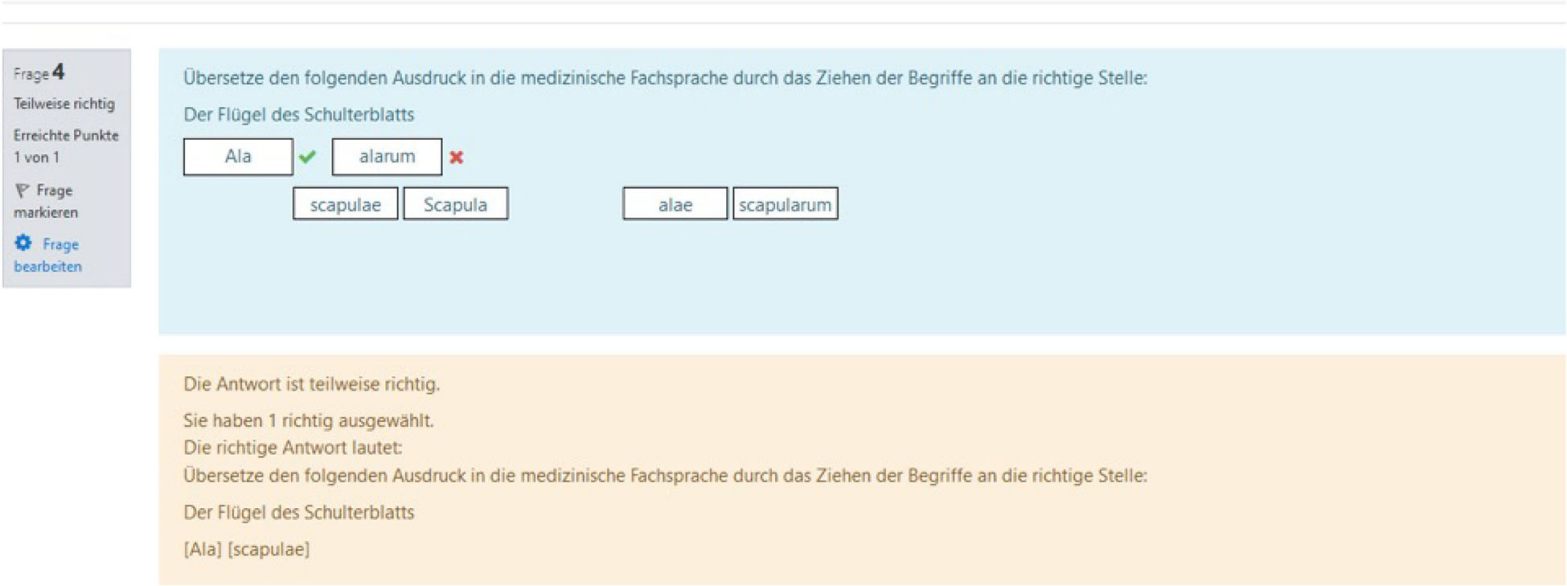
Immediate feedback. Evaluation and feedback on input (here: partially correct)

A focus on the students’ specific needs and their learning environment is achieved, among other things, through the following main features: firstly, using content from anatomy which is taught in the same term as medical terminology and, secondly, using gamification elements. Standard Moodle features (inter alia integrated batches, multimedia integration, track progress) were used to create gamification features. Hence, the TERMInator includes gamification elements, such as progress bars (these show the overall progress within the Modules; Fig. 5), drag & drop (Fig. 6), scoring and certificates (e.g. “TERMI-Novice”, “TERMI-King”, Fig. 7), cascading information and immediate feedback (Fig. 8). As outlined above, certain requirements need to be met in TERMInator before further modules can be accessed. The TERMInator contents are based on those of the textbooks used for teaching [12, 13], the PowerPoint slides presented in the seminars and exemplary tasks from the written exams. The information provided by students in the course evaluations of previous years was also considered while creating TERMInator.

Pilot testing of the first single module took place in the winter term 2018/19, followed by pilot implementation of the complete TERMInator in the winter term 2019/20. An ARSnova link (ARSnova is an Audience Response System which allows an immediate and anonymous feedback) was made available to the students during both stages, pilot testing and pilot implementation, to stimulate immediate feedback regarding any errors or comments that may have occurred. This link was embedded directly into the start page of the Moodle platform. Students were continuously encouraged during the classes to test the TERMInator at home. A short fact sheet containing the essential information on how to find and use the TERMInator was handed out to all students. No further registration was necessary as each student is supplied with a Moodle access by the University Medicine Greifswald.

The TERMInator implementation was comprehensively evaluated. The students were asked to complete an anonymous questionnaire covering aspects of the content, layout and user friendliness of the TERMInator and questions concerning learning preferences. It consisted of sixteen statements that students should rate according to their agreement on Likert scales. One subset of eight questions uses a four-point (“I fully agree” = max., “I agree more with”, “I disagree more with”, “I strongly disagree” = min.) and another subset of eight questions uses a five-point Likert scale (“irrelevant” = min., “not very important”, “important”, “very important”, “most important” = max.). Furthermore, it comprised two questions with dichotomous answers, the opportunity for additional free text comments and one question with a free text answer (see Additional File 1). In addition, the general course evaluations – consisting of two statements, one question and the option of free text commentaries (1. The course had a clear structure. 2. The learning objectives have been achieved. 3. How do you rate the overall course?) – were checked regarding possible changes caused by the TERMInator, as well as the exam results and the overall pass rate of the students. The teaching staff before (winter term 2016/2017, 2017/2018) and during the pilot implementation (2018/2019, 2019/2020) of the TERMInator remained the same (AHS, HB, SS).

## Results

Concerning the general course evaluations, the statement that the course had a clear structure was rated with an overall grade of 2.0 (grade 1.0 relates to the highest grade of agreement) before and after pilot implementation of the TERMInator. The achievement of the learning objectives and the overall assessment of the course changed from grade 3.0 before the pilot implementation to a grade 2.0 after the pilot implementation of the TERMInator. In addition, the students mentioned in their free text answers that they appreciated the new e-learning tool greatly and the TERMInator should be further expanded. The exam results regarding the overall pass rate (pass mark is reached with 60% correct answers) did not change markedly before and after pilot implementation of the TERMInator: Before pilot implementation, 91.4% (winter term 2016/2017) and 95% (winter term 2017/2018) of the students who attended the exam in medical terminology passed it. After pilot implementation, 92.1% (winter term 2018/2019) and 92.8% (winter term 2019/2020) of the students who attended the exam in medical terminology passed it.

**Fig. 9:**
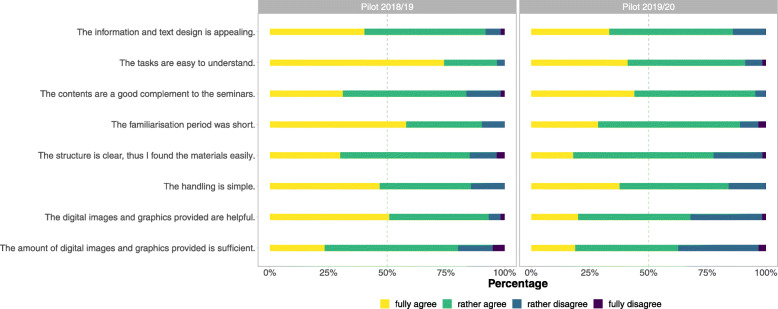
Students’ assessment of TERMInator quality and user friendliness. Relative frequencies of Likert scale responses regarding the quality and user friendliness of the TERMInator for the first (left panel) and the second (right panel) pilot implementation. The horizontal dashed line reflects the median response at each intersection

**Fig. 10:**
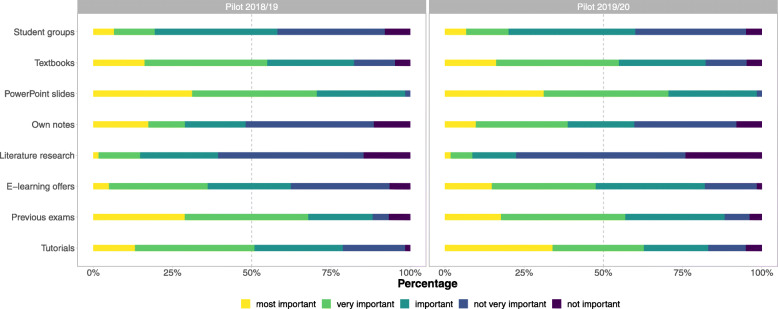
Students’ learning preferences. Relative frequencies of Likert scale responses regarding the learning preferences for the pilot testing (left panel) and pilot implementation (right panel). The horizontal dashed line reflects the median response at each intersection

A total of n = 49 complete and *n* = 16 incomplete TERMInator evaluation questionnaires were analysed in the winter term 2018/19. Another *n* = 48 complete and *n* = 20 incomplete questionnaires for the winter term 2019/20 were included in the analysis. The TERMInator was generally rated very positively, which was also fed back to the lecturers during the classes. According to the TERMInator evaluation questionnaire (Fig. 9), the extent and quality of the images were seen rather critically. However, most students evaluated the overall design positively. The handling of the TERMInator was considered to be very easy and, therefore, very little training time was needed. The tasks were easy to understand and, from the students’ point of view, were a good supplement to the seminar contents. The results also show (Fig. 10) that students’ learning strategies are very different. Although e-learning options were generally rated as very important, tutorials were considered by far the most important learning option.

## Discussion

The TERMInator offers an e-learning environment directed particularly towards the main focuses of the course on medical terminology for human and dental medicine students and can be used free of charge. As the process of implementing additional exercises is based on a mere tabular sheet and has been automatised, a translation to languages other than German could be easily realised.

The benefit of the TERMInator as an e-learning intervention in medical terminology on the part of the students had already become apparent during the pilot implementation, as the students confirmed an increased motivation to practice the contents from the seminars and an increased overall satisfaction with the course. Lecturers of other courses in medical studies, such as Histology [14] and Medical Biology [15], have also shown that game-oriented teaching and learning innovations increased students’ motivation and interest, however, they used explicit gamed-based learning platforms (e.g. Kahoot® [14]) or their own technical solutions (e.g. creating their own website using HTML and JAVA Script [15]) and not existing learning management systems. The prerequisites and possibilities are, therefore, very different and, consequently, hardly comparable. A benefit of the TERMInator on the part of the lecturers could be the digitisation of the exams, which are currently held as a paper and pencil test. Using some adapted tasks of the TERMInator could serve as a basis for the future preparation of an electronic exam. This would represent a substantial facilitation and relief regarding the correction of the tests.

The students are able not only to learn and repeat Latin grammar with the TERMInator but also the other elements of the course (e.g. history of medical terminology). Illustrations can assist the students with remembering the roots of special terms. Furthermore, high-quality images are needed, for example, to practice the body structure, to build the link between medical terminology and anatomy. Nevertheless, an important limitation of the TERMInator is currently the range of images available, which will continue to be a major problem for the further development and expansion of the offer in the future due to limited financial resources and copyright. A challenge to find a suitable format for the exercise of historical content (etymological roots) – which is also a major objective of the course – and to integrate it into the TERMInator according to the overall gamification design also remains.

Gamification is not only a key element for students, but the learning culture is also characterised by increasing mobility [16]. Using the TERMInator as an app on a mobile device – instead of a web browser – is likely to increase both the acceptance and utilisation through an enhanced flexibility.

The development and implementation of the TER-MInator adds to a series of teaching innovations for medical students, such as in the field of epidemics [17] and anatomy education [18]. However, publications on this topic in undergraduate medical studies remain scarce in Germany. One recently published German study showed a significant improvement of motivation and knowledge acquisition in histology through the use of interactive learning software [19].

Regarding the evaluation of the e-learning tool, there are several methodological issues that should be considered. Firstly, the standardised general evaluation is rather unspecific and does not reflect aspects of e-learning at all. However, in the light of changing contemporary teaching in higher education, it should be adapted in order to make it more detailed and include aspects of e-learning. Secondly, passing rates for written exams on medical terminology did not change in the period observed, however, a longer observation period would be necessary or even an experimental design comparing a “TERMInator” with a “non-TERMInator” group for tracking down any potential correlation to the TER-MInator. In this context, potentially relevant socio-demographic and other variables could be collected (e.g. previous knowledge of Latin language). In a first step, the TERMInator evaluation questionnaire could be digitised to be able to compare subgroups within the sample more easily and effectively. This would also allow a retrospective analysis of whether students have used the TERMInator or not and to what extent this is reflected in the exam results. This would, however, entail other considerable difficulties. If the evaluation was conducted after the exam in order to collect the grades, this would result in an (even) lower response rate and potential serious problems concerning data protection and research ethics.

## Conclusions

Medical terminology classes are characterised by heterogeneous learning groups and a high workload within a short time, which can lead to major challenges for the teaching staff. The learning behaviour of individual students shows considerable variation and has changed in recent years, showing a trend towards digitisation. Complementary gamified e-learning offers in medical terminology are promising in view of the students’ different knowledge levels and changing learning behaviour. We encourage educators of medical terminology to take up the challenge of shaping contemporary higher education in a digital form using elements of gamification. Close collaboration with colleagues is advisable to use the available resources across faculties and universities efficiently.

A further development of e-learning tools such as the TERMInator seems promising and should be accompanied by larger and methodologically more intricate evaluation studies. The TERMInator could be implemented at other universities already using Moodle as a learning management system. Combining resources with other sites might improve the TERMInator by enriching its exercise database and, once digitised, offering options for multicentre evaluations.

## Supplementary information

**Additional file 1.** TERMInator Evaluation Questionnaire.

## Data Availability

Data are available on request.
