# Supplementary material for: Gamified E-learning in medical terminology: the TERMInator tool
Source: BMC Med Educ. 2020 Aug 28;20:284. doi: 10.1186/s12909-020-02204-3 (PMC7456391; doi:10.1186/s12909-020-02204-3)
Supplement: Supplementary file 1 — Additional file 1. TERMInator Evaluation Questionnaire. [file 12909_2020_2204_MOESM1_ESM.pdf]

TERMInator Evaluation Questionnaire

1. To what extent do you agree with the following statements?

	I fully agree	*I agree more with*	*I disagree more with*	*I strongly disagree*
The information and text design of TERMInator are appealing.	▭	▭	▭	▭
The tasks are easy to understand.	▭	▭	▭	▭
The contents of the TERMInator are a good complement to the seminars.	▭	▭	▭	▭
Getting familiar with TERMInator costs me a lot of time.	▭	▭	▭	▭
The structure of TERMInator is clear, thus, I found the materials easily.	▭	▭	▭	▭
The handling of TERMInator is simple.	▭	▭	▭	▭
The number of digital images and graphics provided is sufficient.	▭	▭	▭	▭
The number of digital images and graphics provided is sufficient.	▭	▭	▭	▭

2. No technical problems occurred when using the Moodle course TERMInator

I agree ☐

I disagree ☐ --> If problems occurred during the utilisation of the TERMInator, please name/describe them briefly:

____

____

3. I will continue working on the TERMInator as soon as more content is available.

I agree ☐

I disagree ☐ --> What should the TERMInator offer you in order to make it worth your working on it further? ____

4. How do you evaluate the media/methods listed below regarding your personal learning behaviour in medical terminology?

	Irrelevant	*Not very important*	*important*	*Very important*	*Most important*
Student working/learning groups	☐	☐	☐	☐	☐
Textbooks for medical students	☐	☐	☐	☐	☐
Learning with PowerPoint slides from seminar	☐	☐	☐	☐	☐
Learning with one's own notes from the seminar	☐	☐	☐	☐	☐

Further literature search and reading	▭	▭	▭	▭	▭
Utilisation of e-learning offers	▭	▭	▭	▭	▭
Written exams from previous terms	▭	▭	▭	▭	▭
Tutorials held by other students	▭	▭	▭	▭	▭

5. Further suggestions / comments on TERMInator:

____

____
